# Telehealth Barriers and Digital Ageism Experienced by Older Veterans: Formative Ethnographic Study to Inform a Telepharmacy Randomized Trial

**DOI:** 10.2196/79409

**Published:** 2026-04-30

**Authors:** Maria Venegas, Megan B McCullough, Chelsea E Hawley, Judith L Beizer, William W Hung, Lauren R Moo

**Affiliations:** 1 Veterans Health Administration, 200 Springs Road Bld. 70 #128, Bedford, MA, 01730, United States, 1-781-654-7322; 2 Center for Healthcare Organization and Implementation Research, Veterans Health Administration, Bedford, MA, United States; 3 Department of Medicine, Division of Geriatrics, Boston University, Boston, MA, United States; 4 New England Geriatric Research Education and Clinical Center, Veterans Health Administration, Bedford, MA, United States; 5 College of Pharmacy and Health Sciences, St. John’s University, New York, NY, United States; 6 Geriatric Research Education and Clinical Center, James J. Peters VA Medical Center, Veterans Health Administration, Bronx, NY, United States; 7 Department of Geriatrics and Palliative Medicine, Icahn School of Medicine, New York, NY, United States; 8 Department of Neurology, Harvard University, Cambridge, MA, United States; 9 Department of Neurology, Massachusetts General Hospital, Boston, MA, United States

**Keywords:** digital ageism, telehealth, ethnographic research, older adults, telepharmacy, digital divide

## Abstract

**Background:**

As telehealth has become an increasingly common mode of care delivery, older adults may face structural, technological, and interactional barriers that limit their ability to engage with video-based care. Although digital ageism, defined as the presence of age-related stereotypes, lowered expectations, or assumptions about older adults’ technology-related competence, has been described in prior literature, less is known about how such dynamics surface during real-time telehealth encounters and how they may shape participation in technology-focused clinical trials.

**Objective:**

This formative ethnographic study aimed to (1) document real-world barriers encountered by older adults immediately before and during video telehealth visits and (2) inform recruitment and implementation procedures for a subsequent telepharmacy randomized controlled trial.

**Methods:**

We conducted in-home, real-time ethnographic observation of 20 community-dwelling veterans aged ≥65 years participating in pharmacist-led video visits for medication management. Recruitment occurred over approximately 6 to 10 months using mailed invitation letters (>300 sent), supplemented with outbound telephone calls. Data sources included structured field notes completed independently by an in-house anthropologist and the remote clinical pharmacist, as well as observational documentation of previsit preparation, visit navigation, and postvisit reflections. Data were analyzed using qualitative rapid analysis, with iterative team review and triangulation across data sources.

**Results:**

Participants had a mean age of 74 (SD 3.18) years; 19 of 20 (95%) were male, and 18 of 20 (90%) identified as White. All participants completed a video visit with technical support as needed. Structural barriers (eg, broadband access and device availability) and usability challenges (eg, audio-video setup and navigation) were common. Although digital ageism was not a predefined analytical category, age-related assumptions about technology emerged during observation, including participants attributing anticipated or experienced difficulties to age and expressing surprise or pride following successful completion of the visit. These age-related interpretations were analytically distinct from access and usability barriers and were interpreted as manifestations of digital ageism, particularly as internalized age-based expectations.

**Conclusions:**

Formative ethnographic observation provided critical insights into how older adults experience telehealth encounters in real-world contexts and informed adaptations to recruitment and implementation procedures for a subsequent randomized controlled trial. Although digital ageism was not an original study aim, age-related assumptions about technology emerged as an important interpretive factor shaping engagement with video-based care. Incorporating ethnographic methods prior to trial implementation may help identify otherwise overlooked barriers and improve the inclusivity and feasibility of technology-focused clinical research involving older adults.

## Introduction

The COVID-19 pandemic accelerated the adoption of video telehealth as a primary mode of outpatient care delivery, including among older veterans [1-5]. Telehealth, defined by the National Institute on Aging as the use of communication technologies to exchange information and deliver health care services, holds the promise of increasing access to care and reducing logistical barriers while maintaining clinical effectiveness [6]. However, despite widespread interest in telemedicine among older adults, significant obstacles remain [7-12]. Limited broadband access and the high costs of digital devices and internet services—issues that are especially pronounced in rural and low-income communities—restrict many older adults’ ability to engage with telehealth, potentially worsening health inequities [13-18]. Another critical challenge is digital ageism: the assumption that older individuals are less capable of using digital tools [19]. This bias can manifest through technological, structural, and attitudinal barriers [20,21]. Many virtual care platforms are not designed with older users in mind, often featuring small fonts, complex navigation, and controls that are not accommodating for those with motor or visual impairments [13]. In addition, the expectation of digital literacy can alienate individuals who have had limited exposure to technology, leading to frustration and disengagement [12,16,20]. Research within the Department of Veterans Affairs (VA), for example, has shown that while many older veterans are interested in video visits, they often require significant personalized assistance to participate effectively [1,17,22]. Furthermore, some health care providers may default to telephone visits or fail to offer adequate digital literacy support, reinforcing the notion that telemedicine is “not for them” and further discouraging engagement [21,23,24].

Although disparities in video telehealth use among older adults are well documented, a more nuanced understanding of the real-life barriers these individuals face is essential for designing digital tools and platforms that truly meet their needs. To address this gap, we conducted a formative ethnographic assessment of older adults’ experiences during virtual visits with a clinician. This formative ethnographic study aimed to (1) document real-world barriers encountered by older adults immediately before and during a video telehealth visit and (2) inform recruitment and study procedures for a subsequent telepharmacy randomized controlled trial (RCT). Although digital ageism was not a predefined analytical category, age-related assumptions about technology were examined as an emergent interpretative theme.

## Methods

### Study Overview

This study draws on the same formative ethnographic dataset described in our prior publication examining feasibility and practical implementation solutions for pharmacist video visits among older veterans [25]. This manuscript extends that work by focusing on how ethnographic methods can identify and disentangle age-related assumptions from technology familiarity in the design of technology-based clinical trials. The analytical focus and conceptual framing in this manuscript are distinct from those in the prior implementation publication.

### Ethical Considerations

This study was reviewed and approved by the VA Central Institutional Review Board (approval number 19-35). All participants provided informed consent prior to participation. Study staff explained the purpose of the study, procedures, potential risks, and voluntary nature of participation, and participants were informed of their right to withdraw at any time without consequences to their care. To protect participant privacy and confidentiality, all data were deidentified at the time of analysis. Field notes and observational data were stored on secure, VA-approved servers with access limited to authorized study personnel. No personally identifiable information is reported in this manuscript. Participants received a US $50 gift card as compensation for their time.

### Recruitment

Potential participants were identified using an electronic health record–generated list of veterans aged ≥65 years who met the inclusion criteria (eg, ≥2 chronic conditions, no diagnosis of dementia, and at least 1 primary care visit in the prior year). Participants were recruited over approximately 10 months using a combination of mailed invitations and outbound telephone calls. In total, 316 invitation letters were mailed to veterans identified through the electronic health record as potentially eligible. Overall, 64 veterans responded to the mailed invitation; 41 indicated they were not interested in participating, and 23 initially agreed. Of these, 2 veterans subsequently declined enrollment due to personal reasons (ongoing cancer treatment and caregiving responsibilities) and 1 could not be scheduled within the recruitment window; recruitment stopped once the target sample size (n=20) was reached.

To supplement mailed invitations, the study staff conducted outbound telephone calls to veterans who did not respond to the letter. Across mail and telephone contact attempts, veterans either declined participation after learning more about the study or could not be reached (eg, disconnected phone numbers or no response after repeated attempts). In total, 17 veterans were determined to be ineligible due to no longer meeting the study criteria (eg, not currently taking ≥5 medications, recent diagnosis of cognitive decline, or enrollment in a VA satellite clinic). Recruitment ceased once the target sample size (N=20) was reached. All enrolled participants completed the observed telehealth visit.

Our ethnographic assessment used direct observation and structured field notes to capture the real-world experiences of older adults as they navigated a clinical video visit using the VA’s synchronous video platform VA Video Connect (VVC). An experienced anthropologist led this assessment by recruiting and enrolling participants and joining each participant at home before and during their virtual visit.

To participate, all veterans needed internet access at home and a device with a camera and microphone. For those lacking a video-capable device, the anthropologist arranged for the provision of a VA-loaned tablet. The VA’s tablet distribution program was instrumental in bridging the digital divide for veterans with limited access to video care. Veterans receiving a tablet were scheduled for a test visit with a local VA employee, while others received a demonstration from the anthropologist prior to their pharmacist video visit. Before the video visit, the anthropologist provided a video demonstration when needed. Subsequently, the participant and pharmacist connected via VVC.

Ethnographic methods in anthropology involve qualitative techniques—such as semistructured interviews and focus groups—with a unique emphasis on situating specific events within their broader real-life contexts [22,26,27]. Unlike surveys or post hoc interviews, ethnographic methods also rely on in-situ, real-time observations to capture sociocultural, behavioral, and technical challenges that participants may not articulate in interviews [27]. In this assessment, field notes and direct observation notes were critical in documenting the technical, logistical, and emotional dimensions of older adults’ virtual care experiences.

Field notes are detailed, descriptive records that researchers, particularly anthropologists and ethnographers, take during participant observation and interviews [28,29]. We designed a standardized field note form informed by a priori concepts from the Consolidated Framework for Implementation Research (CFIR) [30] and insights from experienced clinical pharmacists (Multimedia Appendix 1). Field notes are generally understood to be primary data collected in the field to document observations, interactions, and insights gained through immersive research methods [28]. Both the in-house anthropologist and the remote clinical pharmacist completed field notes for each participant. The anthropologist’s (in-house) and the clinical pharmacist’s (remote) field notes complemented each other by capturing a range of experiences and challenges, including the following:

- Technical challenges and troubleshooting; difficulties with video connections, microphone access, and screen navigation
- Participant engagement and reactions; variations in comfort, frustration, confidence, or hesitation in using virtual care platforms
- Health care provider interactions; the level of guidance provided, communication tone, and patience in assisting older adults
- Environmental and sociocultural factors; home setup, presence of caregivers, privacy concerns, and distractions

**Figure 1.**
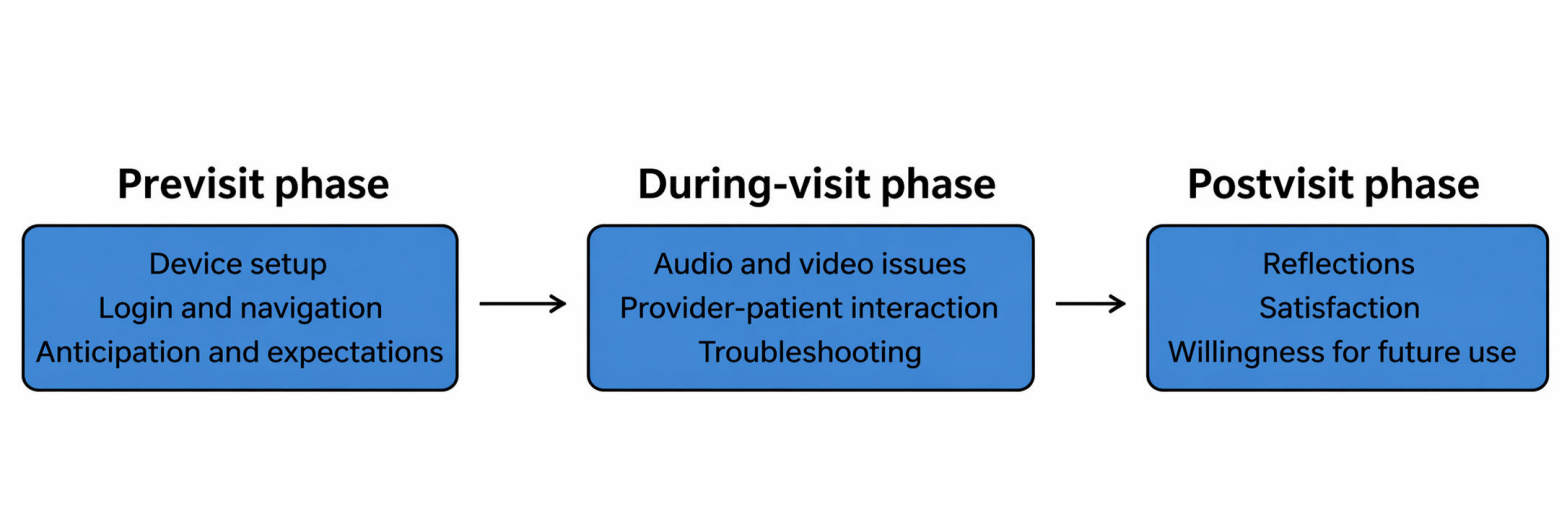
Direct observation in telehealth interactions. This diagram outlines the key stages of direct observation in telehealth research with older adults, including previsit, during-visit, and postvisit phases.

Direct observation is an ethnographic method ideal for studies on naturally occurring behaviors, actions, or events [29]. For example, observation is particularly useful for understanding patients’ experiences because it provides an insider perspective and thus allows researchers to understand end users’ experiences of a problem. Direct observation was conducted before, during, and after the virtual visit session to fully understand the participant’s experience. Key components included the following (Figure 1):

- Previsit observations; how participants prepared (eg, setting up devices and logging in), their anxiety or reluctance related to technology use, and the role of caregivers
- During the virtual care visit; navigation challenges (eg, adjusting volume and enabling the camera), participant-provider interactions, and unanticipated disruptions such as device malfunctions or environmental distractions
- Postvisit reflections; participant feedback on the ease of use, overall satisfaction, willingness to use virtual care in the future, and any adaptive strategies used (eg, using written notes or seeking caregiver assistance)

### Data Analysis

This formative ethnographic assessment was not originally designed to study digital ageism as a primary construct. Rather, the study focused on understanding how older adults navigated real-time telehealth encounters to inform the implementation of a subsequent RCT. Because the study population consisted exclusively of older adults, we anticipated that age-related assumptions about technology, whether expressed by participants, embedded in clinical workflows, or reflected in research procedures, might emerge alongside structural and usability barriers. Data sources included structured field notes completed independently by the in-house anthropologist and the remote clinical pharmacist for each observed visit, as well as observational documentation of previsit preparation and postvisit reflections. All field notes were compiled and organized by participant and visit stage.

We analyzed the ethnographic data (structured field notes and direct observation summaries) using a qualitative rapid analysis approach commonly applied in health services and implementation research, emphasizing matrix-based summarization, team-based interpretation, and iterative refinement of themes [31,32]. This approach is consistent with pragmatic ethnographic methods, including Rapid Assessment Procedure–Informed Clinical Ethnography, which integrates ethnographic observation with rapid, implementation-focused analysis [33].

The rapid qualitative analytic process proceeded in 4 iterative steps. First, field notes were summarized using structured analytic matrices organized by participant; visit phase (previsit, during visit, and postvisit); and focal domains (eg, technical barriers, participant responses, environmental context, and caregiver involvement). This step allowed for systematic comparison across cases while preserving contextual detail. Second, 2 analysts (the anthropologist and the clinical pharmacist) independently reviewed matrix summaries to identify recurring patterns and salient observations. Preliminary themes were generated inductively, with attention to both anticipated implementation challenges (eg, technology access and usability) and emergent phenomena observed during visits.

Third, the 2 primary analysts met weekly over a 6-month period to compare interpretations, refine thematic definitions, and resolve discrepancies through discussion and reference back to the original field notes. These meetings focused on clarifying analytic boundaries, distinguishing overlapping concepts, and ensuring consistency across cases. Fourth, biweekly analytic meetings with a senior qualitative methodologist and a clinical subject matter expert provided an additional layer of review. These meetings were used to challenge emerging interpretations, assess analytic coherence, and confirm that themes were grounded in observational evidence.

Several strategies were used to enhance analytic rigor and credibility [34]. First, triangulation across data sources (anthropologist field notes, pharmacist field notes, and direct observation) allowed for cross-validation of findings. Second, team-based analysis and consensus discussions reduced reliance on a single analyst’s interpretation. Finally, analytic decisions were documented through iterative matrix revisions and meeting notes, creating an audit trail of theme development. This rapid, iterative analytic process enabled the identification of implementation-relevant insights while maintaining methodological transparency and rigor appropriate for ethnographic research embedded within clinical trial preparation.

We distinguished digital ageism from related but distinct phenomena, such as structural barriers to access (eg, broadband availability and device ownership) and usability or design limitations of telehealth platforms. We reserved the term “digital ageism” for age-related stereotypes, lowered expectations, and identity-based assumptions, whether externally imposed or internalized, that shaped participants’ perceptions of their own technological competence and their engagement with virtual care. Consequently, digital ageism was used as an interpretive lens during analysis, rather than as a priori coding framework or outcome.

## Results

### Participant Characteristics

Participants had a mean age of 74 (SD 3.18) years; of 20 participants, 18 (90%) were male, and 18 (90%) identified as White. In total, 11 (55%) participants had completed high school or a General Educational Development test, and 13 (65%) participants reported some prior experience with digital technology (eg, owning or using a smartphone, tablet, or computer). However, experience with video-based platforms was limited for many participants, and assistance was frequently required; 7 (35%) participants reported neither regular access to a video-capable device nor prior experience using such technology and required assistance both before and during the virtual visit. Additionally, 3 (15%) participants reported having access to a device and some experience with technology but nonetheless encountered technical difficulties during the visit that required real-time support.

### Lessons Learned to Refine the Implementation of a Subsequent Telepharmacy RCT

#### Overview

Direct in-home guidance was associated with successful visit completion, and participants frequently expressed comfort with specific tasks (eg, logging in and adjusting audio or video) during or after the visit. These observations informed mitigation strategies incorporated into the subsequent RCT protocol. Common challenges included difficulty navigating devices, audio-visual setup problems, and unstable internet connectivity. These barriers were identified prior to full trial implementation, allowing the study team to develop mitigation strategies such as providing VA-loaned tablets, offering previsit device and platform demonstrations, and delivering real-time troubleshooting support.

#### Identifying and Addressing Barriers Before the Trial

Pretrial qualitative and direct observation helped identify technological and structural barriers that would impact trial retention and data integrity. Direct in-home observations revealed challenges in device navigation, audio-visual accessibility, and broadband connectivity, allowing for proactive solutions such as providing VA-loaned tablets and real-time technical assistance. These observations informed mitigation strategies for the subsequent RCT (eg, previsit platform demonstrations and caregiver involvement strategies).

#### Enhancing Participant Recruitment and Retention

Recruitment was the first opportunity to engage potential participants and build trust. We recruited 20 individuals over approximately 10 months during the COVID-19 pandemic. Despite the challenges of masks and the recommended 6-foot physical distance between parties, participants were accommodating and tolerant of the masking and other restrictions. For some veterans, the anthropologist was the only in-person visitor they had had in months and was greatly welcomed. By meeting patients at their home, we eliminated several barriers to research that homebound older adults face, such as a lack of transportation or mobility issues that limit their ability to participate in hospital-based research. The anthropologist was available via telephone for questions or concerns and was flexible, driving to participants’ homes (sometimes 45 to 75 minutes away in rural locations) to conduct the enrollment and baseline screening when needed, and then returned to observe the in-home pharmacist video visit and conduct the postvisit interview. Often, this occurred immediately following the baseline screening to reduce the burden and infection-related safety risks of scheduling several in-person appointments with the participant. This unconventional approach proved to be successful in reaching out and engaging a population traditionally excluded from clinical and technology-based research and allowed our anthropologist to understand the physical constraints and isolation that many older adults experience on a day-to-day basis.

#### Improving Intervention Implementation

Observational field notes further captured deviations from the planned protocol that would not have been apparent in a traditional trial setting, including extended time needed for technical setup, the role of caregivers in facilitating participation, and the cumulative burden of scheduling multiple appointments. These insights informed refinements to streamline procedures and reduce participant burden. Our ethnographic assessment allowed for adaptation of the intervention before full-scale RCT implementation, such as:

- Adjusting session scheduling to minimize participant burden
- Integrating troubleshooting strategies for common technical difficulties
- Addressing digital literacy gaps before the RCT to improve participant readiness, reduce technical failures, and reduce user frustration during actual trial sessions

By iteratively refining procedures based on our direct observations in the participants’ homes, our ethnographic approach ensured that the RCT was not only scientifically rigorous but also practical and participant-centered. The opportunity to be in the home of the participants ensured that older adults could access the technology and complete the visit and, more importantly, that the anthropologist was able to witness the process by which the video visit was delivered from the patient’s perspective. This step was valuable in understanding how actual implementation differs from planned implementation and in identifying issues important to the transferability of an effective intervention outside experimental conditions [27].

### Analytic Findings

#### Emergent Age-Related Assumptions Shaping Telehealth Engagement

Although digital ageism was not a predefined analytic category, age-related assumptions about technology emerged consistently during observation and participant interaction. We analytically distinguished age-related assumptions from participants’ actual familiarity with technology or prior exposure to digital tools. Although technical challenges and levels of experience varied, age-related assumptions were evident when participants explicitly invoked age to explain anticipated reluctance to engage with technology, rather than when challenges reflected task-specific skills or access alone.

#### Multifaceted Barriers to Video Telehealth Engagement

Older adults faced technological barriers, such as difficulty navigating videoconferencing platforms, small touchscreens, and poor audio-visual accessibility. Structural barriers, including a lack of broadband access, reliance on outdated technology, and the need for assistance with device setup, limited participation in virtual visits. To overcome these hurdles, the anthropologist in our study brought a VA-owned tablet to the homes of those who did not have access to their own device. She also provided a quick demonstration of how to use the VA tablet before the scheduled video visit with the pharmacist. This was especially useful for those who were unfamiliar with touch screens and video call platforms. For participants who did not have access to a device or to reliable broadband internet, the study team took advantage of a national VA program to arrange for a 4G-enabled, VA-issued tablet to be sent to the veteran that included cellular service. Similar task-specific challenges (eg, navigating apps, managing passwords, and enabling audio permissions) were observed among participants with different levels of technology experience and skills, suggesting that difficulty alone did not map cleanly onto age-related assumptions. Furthermore, the anthropologist noted that participants voiced fewer concerns about technology when provided with access to a device and hands-on guided tutorials before and during the virtual visit, when needed.

#### Hands-On Support: Illuminated Barriers and Work-Arounds for Older Adults

Real-time troubleshooting, such as enabling microphone access, adjusting volume settings, or assisting with log-in procedures, was critical in ensuring the successful completion of the video visit with the clinician. We also learned where participants were encountering difficulties and needed support, allowing us to further adapt our protocols. For example, for the RCT, we began asking participants to bring their devices to the consent appointment so that study staff could assist them with tasks such as allowing the VVC app to use the camera and microphone.

#### Direct Engagement With Technology Challenges: Internalized Ageism

In addition to structural and usability barriers, ethnographic field notes documented age-related assumptions that shaped how participants engaged with video visits. Successful completion of the video visit was often accompanied by expressions of relief, appreciation, or surprise, particularly among participants with limited prior experience using video-based care independently. For example, a female participant in her late 70s was at first reluctant to participate in the study because she lacked prior experience with technology. However, when given the opportunity to learn a new skill through hands-on support, she agreed to participate. The participant did not own a smartphone or computer and was skeptical that a clinical video visit would provide the same “whole picture view” as an in-person visit. Prior to the scheduled video visit, the anthropologist provided a short demonstration of how to navigate the tablet, how to set it up, and how to handle it if asked to walk around during the virtual visit. The participant was a little intimidated by the touchscreen modality, as she only owned a flip phone and had never used such a device before. The anthropologist encouraged the participant to practice using the onscreen keyboard, test the video modality, and type her own information into the registration form. During the scheduled video visit, the participant was able to connect and navigate the tablet with no issues. She walked around and showed the clinician her surroundings; she was particularly proud of her home and garden. The participant was able to show the clinician the places where she stored her medications. After the home video visit, the participant was highly satisfied with the experience, indicating that it felt like an in-person visit.

#### Personalized Adaptations for Older Adults

For those participants with no digital technological skills, the anthropologist demonstrated how to use the video app before the actual clinical video visit and often had to assist the participant during the video visit by solving small issues such as enabling the video app, increasing the volume, or resolving Wi-Fi connection issues. However, even for those with more digital literacy, telemedicine and video telehealth policies often assume that older adults can seamlessly transition to digital platforms, without accounting for the need for additional training or technical support for practical issues.

For example, 1 participant had an older desktop without a camera that he used for email but had access to an iPad tablet with an integrated camera, which he had never used for email. The anthropologist sent instructions on how to add an email account to his iPad. However, as is common with many older adults, his internet service provider was also his email provider. The participant attempted to add his email to his iPad but gave up, stating, “I’m too old for this.”

Adding an email account to a new device can be challenging for older adults who do not have much technical experience (even for those who are tech-savvy, as the instructions can be lengthy and difficult to follow). Similarly, email technology is not always up to date, which introduces potential problems: the interface may be difficult to use or may not work with web browsers. We used FaceTime (Apple Inc) briefly as a practice video call to familiarize the participant with camera and audio controls; the pharmacist visit itself occurred via VVC. The anthropologist assisted with enabling the video app and manually toggled the camera switch when needed. Participants described feeling respected and listened to during pharmacist interactions and emphasized the value of being able to speak “face to face” while accessing medications in their home. These responses reflected shifts in how participants evaluated their own capacity to engage with telehealth and their ability to speak openly with a pharmacist about medication-related questions.

#### Sociocultural and Environmental Factors Influence Virtual Care Success

Home setup challenges (eg, lack of private space, poor lighting, or technical constraints) impacted the quality and comfort of virtual care interactions. Participants with higher digital literacy or prior exposure to technology were more likely to engage diligently in virtual care, while those with minimal digital experience required more ongoing support. The anthropologist noted that some participants changed their environment by figuring out ways to prop up a device to be hands-free such as stacking books. Ethnographic observation further revealed that negative prior experiences with technology shaped participants’ expectations and behaviors. Some participants expressed distrust of digital technologies due to concerns about privacy, hacking, and fraud, citing repeated scam calls or prior financial compromises. These concerns influenced device use practices, such as disabling or covering cameras, frequently changing passwords, or limiting technology use to specific tasks, such as email or banking. Thus, for these participants, reluctance to use video visits was rooted not only in technical skill but also in perceived risk and vulnerability. Family and caregiver involvement played a crucial role in supporting digital engagement. Some participants reported having their spouses, grandchildren, or children assisting with technical support when engaging with technology in general, as one participant noted, “You know who I learned that from? My granddaughter. She knows how to do this.”

## Discussion

### Principal Findings

This formative ethnographic study examined how older adults engaged with video-based telehealth encounters in real-world settings to inform recruitment and implementation strategies for a subsequent telepharmacy randomized clinical trial. Through in-home observation conducted before, during, and after pharmacist video visits, we identified a range of structural, technological, and sociocultural factors that shaped telehealth engagement. Through direct observation, we gained insight into the disconnect between technological design and user needs, highlighting where telehealth platforms fail to accommodate older adults and how digital ageism manifests in real-world health care settings. These insights directly informed the development of recruitment and technology troubleshooting methods for the subsequent RCT focused on pharmacist video visits for medication management among older adults. In addition, although digital ageism was not a core focus of our study, age-related assumptions emerged as an important interpretative factor that influenced how participants anticipated, experienced, and evaluated their own video visits.

Consistent with prior work, our findings underscore that telehealth engagement among older adults cannot be understood only in terms of individual technical skills or motivation [4-7,9-15]; our in-depth observational approach uniquely highlights both the facilitators and obstacles encountered before and during video visits. Structural barriers such as broadband access, device ownership, and email access intersect with sociocultural factors, including household layout, rurality, and caregiver presence. In-home observation allowed us to identify challenges that would not have been apparent through remote assessment alone, such as difficulty transporting devices within the home, concerns about privacy, and other physical limitations.

Family members frequently served as informal technology facilitators, assisting in device setup, account management, and troubleshooting. This finding highlights the importance of considering digital literacy programs tailored to older adults’ needs as potentially dyadic, including community-based initiatives and caregiver-led tutorials [25,35].

Age-related assumptions about technology surfaced during observation and participant interaction. Participants’ reluctance to engage with video visits was attributed to older age and lower technical experience. Although successful completion of video visits was frequently accompanied by expressions of surprise or pride, suggesting that telehealth encounters functioned as moments in which age-related self-perceptions were reinforced. These patterns can be further understood through a sociopsychological identity framework, which conceptualizes age and generational status as socially constructed identities rather than fixed deficits. As described by Lee and Hartanto [36], individuals’ engagement with technology is shaped not only by skills or access but also by identity-relevant meanings, stereotypes, and expectations associated with belonging to an age group. From this perspective, observed changes in confidence reflect changes in how participants interpreted their own age-related technological competence rather than simple resolution of usability barriers.

It is important to note that not all age-related considerations observed in this study should be interpreted as manifestations of ageism. Some adaptations, such as preparing for visual, hearing, or mobility limitations, enabled and supported successful participation in the study. These practices reflect responsive design rather than ageist assumptions. The analytical challenge lies in distinguishing between accommodations that enhance inclusion and assumptions that lower expectations or discourage engagement. Although similar technology challenges might arise in younger populations with limited technology exposure, the tendency to attribute difficulty to age identity and the meaning attached to this appeared to be specific to older adults’ experiences in this context.

Our findings suggest that incorporating ethnographic methods prior to trial implementation can surface otherwise overlooked barriers and facilitators to telehealth engagement among older adults. In-home observation provided actionable insights that informed protocol refinements, recruitment strategies, and support mechanisms for the subsequent randomized trial. Broadly, these methods may help ensure that technology-focused clinical research does not inadvertently exclude older adults by assuming access, skills, or comfort with digital tools.

### Limitations

As with most qualitative research, our findings are not broadly generalizable to populations at large. The sample size was small and drawn from veterans willing to participate in a formative telehealth study and thus does not reflect the demographics of older adults in the United States. Given the technology acceptance and uptake during the COVID pandemic, the individuals enrolled in our study likely had additional motivation to learn to use video technology and thus participate in our study. Additionally, as this was a formative qualitative study, findings reflect observed experiences and reported perceptions rather than measured intervention effects. Finally, our hands-on ethnographic approach to recruitment and participant engagement with technology may not be feasible for all studies, especially those that are larger and time limited.

### Conclusions

We conducted a focused ethnographic assessment of 20 older individuals engaging in a virtual clinical visit. We illustrate that the application of ethnographic methods to identify and reduce critical barriers to participation and retention in complex health interventions among older adults can yield critical information to improve an RCT. We offered critical insights into how older adults experience telehealth encounters in everyday contexts and revealed the interplay of structural barriers, social support, and age-related self-perceptions. Although digital ageism was not an initial focus of this study, age-related assumptions about technology emerged as a factor shaping engagement with virtual care. Integrating ethnographic approaches and methods into telehealth research and implementation efforts may help improve inclusivity, feasibility, and responsiveness for older adult populations.

## Supplementary material

10.2196/79409 Multimedia Appendix 1 Structured field note template used to guide ethnographic observations during telehealth visits.

## Abbreviations

CFIR: Consolidated Framework for Implementation Research
RCT: randomized controlled trial
VA: Department of Veterans Affairs
VVC: VA Video Connect

## References

[1] Padala KP, Wilson KB, Gauss CH, Stovall JD, Padala PR (2020). VA video connect for clinical care in older adults in a rural state during the COVID-19 pandemic: cross-sectional study. J Med Internet Res.

[2] Weldon AL, Hagemann L (2023). Telehealth use and COVID-19: assessing older veterans’ perspectives. Psychol Serv.

[3] Albritton J, Ortiz A, Wines R (2022). Video teleconferencing for disease prevention, diagnosis, and treatment: a rapid review. Ann Intern Med.

[4] Predmore ZS, Roth E, Breslau J, Fischer SH, Uscher-Pines L (2021). Assessment of patient preferences for telehealth in post-COVID-19 pandemic health care. JAMA Netw Open.

[5] Hogan J, Amspoker AB, Walder A, Hamer J, Lindsay JA, Ecker AH (2022). Differential impact of COVID-19 on the use of tele-mental health among veterans living in urban or rural areas. Psychiatr Serv.

[6] Telehealth: what is it, how to prepare, is it covered?. National Institute on Aging, National Institutes of Health.

[7] Frydman JL, Li W, Gelfman LP, Liu B (2022). Telemedicine uptake among older adults during the COVID-19 pandemic. Ann Intern Med.

[8] Samson LW, Tarazi W, Turrini G, Sheingold S (2021). Medicare beneficiaries’ use of telehealth in 2020: trends by beneficiary characteristics and location. https://aspe.hhs.gov/reports/medicare-beneficiaries-use-telehealth-2020.

[9] Bhatia R, Gilliam E, Aliberti G (2022). Older adults’ perspectives on primary care telemedicine during the COVID-19 pandemic. J Am Geriatr Soc.

[10] Choi NG, DiNitto DM, Marti CN, Choi BY (2022). Telehealth use among older adults during COVID-19: associations with sociodemographic and health characteristics, technology device ownership, and technology learning. J Appl Gerontol.

[11] Donaghy E, Atherton H, Hammersley V (2019). Acceptability, benefits, and challenges of video consulting: a qualitative study in primary care. Br J Gen Pract.

[12] Levy H, Janke AT, Langa KM (2015). Health literacy and the digital divide among older Americans. J Gen Intern Med.

[13] Mao A, Tam L, Xu A (2022). Barriers to telemedicine video visits for older adults in independent living facilities: mixed methods cross-sectional needs assessment. JMIR Aging.

[14] Thomas-Jacques T, Jamieson T, Shaw J (2021). Telephone, video, equity and access in virtual care. NPJ Digit Med.

[15] Choi EY, Kanthawala S, Kim YS, Lee HY (2022). Urban/rural digital divide exists in older adults: does it vary by racial/ethnic groups?. J Appl Gerontol.

[16] Moo LR, Schwartz AW (2021). The urgent need for rigorous studies of telehealth for older adults who are homebound. JAMA Netw Open.

[17] Moo L, Nguyen A, Pugh K, McLaren J, Shirk S, O’Connor M (2022). Technical supports to bridge the digital divide for older adults participating in telemedicine. Innov Aging.

[18] Haimi M (2023). The tragic paradoxical effect of telemedicine on healthcare disparities- a time for redemption: a narrative review. BMC Med Inform Decis Mak.

[19] Chu CH, Nyrup R, Leslie K (2022). Digital ageism: challenges and opportunities in artificial intelligence for older adults. Gerontologist.

[20] Chu C (2023). Digital ageism and its implications for older adult inclusion. Innov Aging.

[21] Seifert A, Cotten SR, Xie B (2021). A double burden of exclusion? Digital and social exclusion of older adults in times of COVID-19. J Gerontol B Psychol Sci Soc Sci.

[22] Smith-Morris C, Lopez G, Ottomanelli L, Goetz L, Dixon-Lawson K (2014). Ethnography, fidelity, and the evidence that anthropology adds: supplementing the fidelity process in a clinical trial of supported employment. Med Anthropol Q.

[23] Leff B, Ritchie CS, Rising KL, Cannon K, Wardlow L (2025). Addressing barriers to equitable telehealth for older adults. Front Med (Lausanne).

[24] Ladin K, Porteny T, Perugini JM (2021). Perceptions of telehealth vs in-person visits among older adults with advanced kidney disease, care partners, and clinicians. JAMA Netw Open.

[25] Hawley CE, Wagner C, Venegas MD (2024). Connecting the disconnected: Leveraging an in-home team member for video visits for older adults. J Am Geriatr Soc.

[26] Morgan-Trimmer S, Wood F (2016). Ethnographic methods for process evaluations of complex health behaviour interventions. Trials.

[27] O’Cathain A, Thomas KJ, Drabble SJ, Rudolph A, Hewison J (2013). What can qualitative research do for randomised controlled trials? A systematic mapping review. BMJ Open.

[28] Sanjek R (1990). Fieldnotes: The Makings of Anthropology.

[29] Fix GM, Kim B, Ruben MA, McCullough MB (2022). Direct observation methods: a practical guide for health researchers. PEC Innov.

[30] Damschroder LJ, Reardon CM, Widerquist MAO, Lowery J (2022). The updated Consolidated Framework for Implementation Research based on user feedback. Implement Sci.

[31] Gale NK, Heath G, Cameron E, Rashid S, Redwood S (2013). Using the framework method for the analysis of qualitative data in multi-disciplinary health research. BMC Med Res Methodol.

[32] Hamilton AB, Fix GM, Finley EP (2024). Pragmatic Healthcare Ethnography: Methods to Study and Improve Healthcare.

[33] Palinkas LA, Zatzick D (2019). Rapid Assessment Procedure Informed Clinical Ethnography (RAPICE) in pragmatic clinical trials of mental health services implementation: methods and applied case study. Adm Policy Ment Health.

[34] Abraham TH, Finley EP, Drummond KL (2021). A method for developing trustworthiness and preserving richness of qualitative data during team-based analysis of large data sets. Am J Eval.

[35] Jacobs JC, Greene L, SooHoo S, Slightam C, Gujral K, Zulman DM (2025). The role of social support in bridging the digital divide for older veterans. Med Care.

[36] Lee D, Hartanto A (2025). A cultural identity approach to the generational divide. New Ideas Psychol.

