# Supplementary material for: Telehealth Barriers and Digital Ageism Experienced by Older Veterans: Formative Ethnographic Study to Inform a Telepharmacy Randomized Trial
Source: JMIR Aging. 2026 Apr 30;9:e79409. doi: 10.2196/79409 (PMC13135888; doi:10.2196/79409)
Supplement: Multimedia Appendix 1 [file aging-v9-e79409-s001.docx]

***Site Visit:***

**Team Member:** **Participant ID #**

| **Devices used for encounter** | | |
| --- | --- | --- |
| Tablet/IPAD  Smart Phone  Personal use laptop  VA loan  Broadband (WIFI, 3G, 4G)  Notes:  **Health and Cognition**  Hearing issues  Visual issues  Requests for louder/rephrase  Mobility issues  Need for technical assistance  Describe: | **Logistics**  Where did Veteran go within home  Kitchen  Bathroom  Bedroom  Describe:  **Facilitators:** | **Barriers:**  **Communication and psych variables**  Anything ignored/missed by clinician  Ignored/missed by Veteran  Evidence of discomfort (tech or people in home)  Apologies (mess/clutter)  Other _ |
| **Visit Steps completed:** Setup:       Intro:       Identification of meds/regimes:  Description/visualization of how to take meds:  Clarify instructions:  Questions and closing:  Survey:       Interview : | | |
| **Adaptations (any changes or new strategies to the intervention) :**  **General Observations (Contextual factors; Social Determinants of Health; interpersonal factors):**  **Key Take-Aways (notes of significance):** | | |
